# Locating the ‘culture wars’ in laboratory animal research: national constitutions and global competition

**DOI:** 10.1016/j.shpsa.2021.08.010

**Published:** 2021-10

**Authors:** Gail Davies

**Affiliations:** Department of Geography, University of Exeter, Amory Building, Rennes Drive, Exeter, EX4 4RJ, UK

**Keywords:** Animal research, Licensing, Standards, Regulation, Constitution, International comparison

## Abstract

The increasingly global scope of biomedical research and testing using animals is generating disagreement over the best way to regulate laboratory animal science and care. Despite many common aims, the practices through which political and epistemic authority are allocated in the regulations around animal research varies internationally, coming together in what can be identified as different national constitutions. Tensions between these periodically erupt within the laboratory animal research community as a ‘cultural war’ between those favouring centralised control and those advocating local flexibility. Drawing on long-term engagement with key events and actors in these policy debates, I propose these national differences in the constitution of animal research can be understood through the intersection of two key variables: i) the location of institutional responsibility to permit research projects and ii) the distribution of epistemic authority to shape research practices. These variables are used to explain the development of different policy frameworks in the UK, Europe, and the USA, and identify where there is convergence and divergence in practice. Concluding, I suggest the way these approaches are combined and enacted in different countries reflects different national civic epistemologies, which are coming into conflict in the increasingly global networks of laboratory animal science.

## 1 Introducing a ‘culture war’

The UK has now left the European Union (EU) but has yet to redefine its ongoing regulatory relationship with scientific research in Europe, the United States of America (USA), or other countries like Singapore. The specific legal frameworks around animal research, which the UK has shared with the EU since 2013, have been transposed into UK law, meaning current regulations will continue to apply to this area of research. However, as Hilgartner et al. argue, the constitution of political and epistemic authority to do something like animal research “encompasses but is not limited to the formal, legal realm” (2015, p. 7). Social permissions to carry out research on animals change over time through the clarification of guidelines, the application of harm-benefit review, and the licensing of projects. Scientific research practices shift through the setting of standards, the interpretation of protocols, and the intervention of experts who oversee research. Even without formal legal changes, there are many potential decisions involved in the re-constitution of UK animal research. The UK has opportunities to realign its research practices with international collaborators in the USA, Europe, or Asia, but it also faces challenges given differences in the way animal research regulations are already constituted globally. It is not yet clear which ways UK regulation might shift to align itself with different countries and, given international complexities in the policies governing animal research, it is also hard to predict the practical consequences of change.

In this paper, I develop a framework for understanding international differences in the regulation of animal research, focusing on discussion of policy and practice in the UK^1^ and the USA over the last 20 years. I introduce key regulatory differences between these two countries and suggest these reflect their broader political constitutions, that is “the patterned ways in which societies allocate powers, rights, burdens and entitlements” (Hilgartner et al., 2015, p. 7). Demonstrating how differences in the regulation of animal research are embedded in national constitutions indicates how challenging they are to transform. Furthermore, locating these differences within a longer history of international competition over regulation indicates the stakes involved in changing regulation: whether connecting to emerging centres of scientific research or, as in the case of the UK, disconnecting from established practices of research governance. These discussions are thus of interest to scientists and regulators in the UK. They are also of relevance to the growing body of social scientific research on laboratory animals, which is seeking to understand how science, ethics, and welfare are enacted in relation to policy practices (Davies et al., 2018). Care in laboratory animal science, as in other policy contexts, is dependent on a complex repertoire of technical, material, and bureaucratic practices (Asdal, 2015; Asdal & Druglitro, 2017, pp. 66–84; Gill et al., 2017). As further national studies emerge of how administrative techniques are used to manage conflicting issues around animal use (Druglitro, forthcoming), it is useful to look at the broader contexts in which these techniques are coming into conflict themselves.

These regulatory differences may seem obscure, but some are so significant they have been characterised as a ‘cultural war’ by those who work in the field. This term was used by someone active in debates about laboratory animal use and care in the USA, when we talked during an intense period of international regulatory review in the early 2010s. They suggested:

*“The debate about harmonising between Europe and Canada and North America is a waste of time, only because in all three situations the animals appear to be doing well enough. Tweaking the size of the cage, or whatever other variables you want to tweak, is going to have zero or minimal impact on the welfare of the animal, unless you can come up with some new metrics that are scientifically based and not just on our impressions of how the animals may feel better. It's a very cold American attitude. […] ICLAS*^2^*and ICH*^3^*and harmonisation and some of these other cultural wars, to me are silly.”*

The US *Guide for the Care and Use of Laboratory Animals* (hereafter, *The Guide*) and those elements of UK policy and practice incorporated into EU Directive 2010/63 represent two poles of this culture war. In this period, the Washington-based Institute for Laboratory Animal Research (ILAR) had just published revisions to the 8th edition of *The Guide* (National Research Council, 2011b), following a seminar exploring Animal Research in a Global Environment (National Research Council, 2011a). And in Europe, there were a series of meetings leading up to the EU Directive 2010/63, which was enacted in 2012 and required members states to implement legislation through their national laws in 2013. Many people involved in drafting these documents attended both sets of meetings and both processes of regulatory review had international implications. The EU Directive established a regulatory framework for governing animal research across the EU. This closely followed key aspects of UK legislation, including the centrality of efforts to replace, reduce, and refine the use of animals in research (the 3Rs), which had been incorporated into the UK Animals (in Scientific Procedures) Act since 1986. *The Guide* outlined requirements for publicly-funded animal research in the USA, but also has global reach as it is used in the international accreditation processes run by the US-based Association for Assessment and Accreditation of Laboratory Animal Care International (AAALAC, n.d.).

In what follows, I draw on long-term engagement with key events and actors in these policy debates, integrating empirical insights from research and committee experience with the academic and policy literatures on animal research.^4^ My argument proceeds in three main stages. In section 2, I develop a framework for moving beyond the complexities of national regulations in animal research to comparing different constitutions. I draw on literature in science and technology studies (STS) and regulation to propose many international differences can be explained by looking at the intersection of two key variables in national governance: i) *the location of institutional responsibility to permit research* and ii) *the distribution of epistemic authority to shape research and care*. In section 3, these are applied to explain key differences in policy and practice in the UK and USA. The importance of which institutions have ethical responsibility to permit animal research is illustrated through licensing policies in the UK and USA. The distribution of what Valverde (2003) calls ‘epistemic authority’ in practice is demonstrated through the operation of engineering and performance standards for animal care in UK/Europe and the USA. In section 4, I explain why these policy differences exist and locate them within what Jasanoff (2005) calls the different civic epistemologies of the UK and USA. This leads to some convergence in the practices of animal research but has the potential to lead to conflict and gaps when extended elsewhere. Turning briefly to how competition between UK and US regulations is playing out in Singapore, I close with some reflections on the enduring importance of national constitutions within an increasingly global science.

## 2 Developing a framework for comparing regulations

### 2.1 Changing geographies of science and regulation

National regulations for animal research not only need to balance the interests of science, industry, publics, and animal protection, but also consider how to manage scientific collaborations, set standards, share research data, and protect international trade. As one review suggests this involves negotiating a complex mosaic of geography, law, and science:

“The oversight of animal care and use occurs through a wide variety of local, national, and international mechanisms, some based on legislation [the European Union (EU)]; others on peer review or other forms of nonlegislated oversight (Canada) and yet others on a combination of legislated and nonlegislated oversight (United States). This patchwork of mechanisms can cause problems, given the global nature of science.” (Demers et al., 2006, p. 700)

These complexities can be hard for even policy-makers and stakeholders to grasp. They are distributed across national and international documentation that detail licensing procedures and accreditation rules, define standards and indicators, provide codes of practice, offer guidelines and guidance, proffer concordats and declarations, and seek to anchor the principles of the 3Rs internationally. These documents, a selection of which are discussed in more detail below, both explain what is required by national laws and often extend what is mandated by national legislation by applying local regulations to overseas collaborations.

There is now consensus around many key points. The role of the animal use and care committee,^5^ whether legislated or non-legislated, is required within many national regulations, often including a role for lay people (Mohan & Huneke, 2019; RSPCA & LASA, 2015). Laboratory animal suppliers are regulated in many countries and consideration of the 3Rs is increasingly mandated within international policy, though this is still uneven in practice (Bayne et al., 2015; Turner et al., 2015). There is growing global attention to the other Rs of reproducibility, replicability, and rigour (Baker et al., 2014; Canadian Council on Animal Care, 2019; Macleod & Mohan, 2019). And there are ongoing efforts to understand the global landscape of animal research and co-ordinate better science and welfare within the research community (Bayne & Turner, 2019). However, many significant differences remain, which tend to erupt when discussion turns to the international harmonisation of standards (Demers et al., 2006). Divergent attitudes to centralised standards are rooted in different political cultures and in competition about who might win or lose in the growing global knowledge economy (Dietz et al., 2018; Doezema & Benjamin Hurlbut, 2017). These pressures are accelerating with the growth of risk-based approaches to regulation as government budgets tighten across Europe and North America. Developing a framework for characterising differences in the governance of animal research has to encompass national cultures, international competition, and regulatory pressures. I review each of these briefly below.

Sheila Jasanoff has worked for over twenty years on the entanglements between science, the state, and political culture, primarily in the USA, the UK, and Germany (Jasanoff, 2004, 2005, 2007; Jasanoff & Hurlbut, 2018). Her work illustrates how national political cultures, and what she calls ‘civic epistemologies’, continue to matter in the context of the globalisation of scientific research, the ‘fracturing of the authority of nation-states’ (2005, p.14), and the need to renew social contracts with science. Jasanoff is interested in how science and reason operate in public life and the different constitutional forms through which liberal democracies manage the risks of new technologies, like genetic modification and gene editing (Jasanoff & Hurlbut, 2018). Her term ‘civic epistemology’ refers to the ways knowledge is ‘presented, tested, verified and put to use in public arenas’ (2005, p.258) and draws attention to how authority is distributed, how objectivity is performed, and how publics are involved in the production of new social relations with science and technology. Her approach to the co-construction of political and epistemic cultures has been highly influential in STS. Her work has been used to explain the divergent geographies of a wide range of technological developments, such as embryonic stem cell research (Yoon et al., 2010), as well as to explore differences in the national cultures of animal research by Friese et al. (2019).

A different strand of work on the changing geographies of science starts with the management of international scientific networks and technological zones (Barry, 2001, 2006), rather than the constitution of national governance. This reflects the growing emphasis in neoliberal science on international investment strategies (Lave et al., 2010). The contemporary biosciences are increasingly recognised as part of a global economic system, with established research centres in Europe and North America joined by emerging knowledge economies in China, India, Singapore and elsewhere (Leadbeater & Wilsdon, 2007). Scientific research is connected internationally through the intensification of transnational collaboration and the networks of global pharmaceutical firms. These changing geographies of science are motivated by the value of international cooperation and the drive for geopolitical competition, and are underpinned by different strategies for state and private innovation (Salter, 2009; Sunder Rajan, 2012). In this context, an interest in comparison is not only driven by academic curiosity, but also emerges as a national innovation strategy as governments study regimes of scientific governance elsewhere to borrow effective policies (Peck, 2011) and seek competitive gains through exploiting their relative advantage (Salter, 2009).^6^ Both charting and navigating this international landscape is particularly challenging in laboratory animal research as it incorporates different kinds of scientific practice and regulatory control in basic research, pharmaceutical development, product testing, animal breeding and supply, all of which vary internationally (Davies, 2013; Niemi & Davies, 2016).

The third element accelerating change within the international landscape of animal research is the growth of risk-based regulatory processes. This has been a prominent development across UK regulation and public management (Black, 2005) and can be seen in the introduction of risk-based inspections within the UK's Animals in Science Regulation Unit (ASRU, 2020) as well as the management of farm animal welfare through audit practices, metrics, and behaviour change (Escobar & Demeritt, 2017).^7^ Divergent national responses to the international rise in risk-based approaches have been examined in work by Geographer David Demeritt and others (Demeritt, 2000; Demeritt et al., 2015; Rothstein et al., 2017). They caution against simply collecting case studies of regulatory differences and have developed a systematic way for comparing how the location of regulatory responsibility (primarily between central and regional or local governance) effects how risk-based approaches to governance are used in practice across different countries. Their work, spanning food to floods, has inspired this attempt to develop a framework for animal research that can encompass key differences between UK/EU and US regulation and be used to explore regulatory approaches elsewhere. Drawing on their work, I propose two variables can be used to characterise many key aspects of the international debate around laboratory animal use and care: the *location of institutional responsibility to permit research* and the *distribution of ‘epistemic authority’ to shape research and care* with animals.

### 2.2 Introducing two key variables

The *location of institutional responsibility to permit research* is relatively easy to identify as it is given by law or explained in guidelines. The allocation of this responsibility in different countries indicates who is seen as the ultimate ethical decision-maker around animal research and reflects how far the adjudication between enabling research and protecting animals is seen as the role of the state or assigned to another body or person. Most countries that have laws to protect animal welfare have a concomitant process by which certain people, in certain places, and for certain permitted purposes can be allowed to carry out research on animals. These permit something to happen to an animal, which is not in the best interests of that animal, so would generally be considered to be animal cruelty through law. The STS literature has indicated how animal bodies are transformed into experimental objects through the outcome of legal arguments (Asdal, 2008) and national licensing (Druglitro, forthcoming). However, there has been less attention to how the responsibility to permit or licence animal research varies internationally and why.

When this responsibility is allocated to the state, the decision on whether to permit animal research resides with public bodies, and scientists must submit their justifications to the appropriate government department. There are a number of countries where this responsibility to permit research is centrally located. This includes the UK, where responsibility resides in the licensing system of ASRU, as well as Singapore and some European Countries (Retnam et al., 2016; Vasbinder & Locke, 2016). Other European countries, such as Germany, allow regional governments to give permissions for animal research. These approvals function in a similar way to those given by a centralised state but allow some regional variation. Yet other countries, such as the USA, have devolved power to permit research to the level of local institutional animal care and use committee. The location of the responsibility to permit animal research reflects divergent visions of what the state is for and empowers different institutional forms. The involvement of the state is seen as essential for safe-guarding animal welfare in some jurisdictions, including in the UK and much of Europe, but is often constructed as inefficient and ineffective in this role in others, such as North America.^8^

Other aspects of regulation flow from the location of institutional responsibility to permit research. Designated authorities need to have in place competent actors to discharge these responsibilities and complete processes of record keeping, audit, and inspection. The challenge of establishing and maintaining new systems to oversee research can be seen in the 2018 review of how EU Directive 2010/63 was operating across member states. Six member states^9^ were contacted about legal shortcomings in areas such as provisions on inspections, staff competences, record keeping, and the use of penalties.^10^ Challenges also emerge when research is authorised locally, where they may be differences in local policy and practice, which still need to co-ordinate across sites in order to ensure compliance with the requirements of public funders, transnational companies, professional societies, and international accreditation through bodies such as AAALAC. Each regulatory policy thus creates pressures on how best to organise the people, budgets, and processes to facilitate research and protect animals. This may lead to some convergence in practice. Central regulators may use risk-based approaches to do things like triage attention and oversight, whilst local bodies may rely on externally set regulations to guide their work. However, the outcomes then depend on how authority is distributed to speak in impactful ways to shape practices of animal research and care.

The second variable I want to introduce is the *distribution of what* *Valverde (2003)* *calls ‘epistemic authority’ in the practices of research and care*. This draws attention to who has authority for managing protocols and protecting animal welfare during research practices. Simply comparing the institutional responsibility for ethical review is insufficient to account for the international differences in laboratory animal research in part due to the “diverse modes of knowledge” operating within these “legal complexes” (Valverde, 2003, p. 11). A further key variable is whether epistemic authority to influence the operation of animal care and use is distributed across a wide network of people and materials or concentrated in a few roles and infrastructures. Such epistemic authority defines who is able to speak out, who is listened to, and who is empowered to act, and over what. Epistemological power can be concentrated in the hands of certain scientific experts, such as the role of the professional veterinarian within the IACUC in the USA, or it can be more distributed through the web of named roles that constitute the system of UK regulation and care (Kirk, forthcoming). The distribution of epistemic authority across government regulators, institutional veterinarians, scientific researchers, lay members, and others involved in the implementation of legislation varies across national contexts independently to the institutional responsibilities for permitting animal research outlined above. This introduces flexibility by extending discretion in the operation of animal research. It also introduces variability. Authority may be understood and exercised in different ways, whether protecting against risks to animal welfare or perceived risks to furthering scientific innovation.

In what follows, I use these two variables to examine key differences between the regulation of animal research in the UK and the USA. These countries feature frequently in the literature due to the global position of US science and medicine, and the extensive nature of AAALAC accreditation, and the long history of animal research regulation in the UK, which informed the development of the EU Directive. They also occupy contrasting positions in the key dimensions outlined above (see Table 1).

**Table 1:** Two key variables in the regulation of animal research.

		Institutional responsibility to permit research	
		centralised	distributed
Epistemic authority to shape research and care	concentrated		e.g. USA
	distributed	e.g. UK	

Institutional responsibility is highly centralised in the role of ASRU in licensing animal research in the UK, whilst local institutional animal care and use committees have responsibility for permitting much research in the USA. However, epistemic authority for animal welfare is widely distributed through named institutional roles in the UK and more concentrated in the role and high professional standing of the vet in the USA. The so-called ‘cultural wars’ around animal research revolve in part around this stark contrast evident in the complex licensing system in the UK and the exclusion of legal protection for many laboratory rodents in the USA. Depending on your perspective, the UK is perceived as either the most complete system of regulation in the world or the most cumbersome, and the USA the most efficient or deficient. In the next section I start my exploration of these differences there.

## 3 Identifying differences in policy and practice

### 3.1 Who licences animal research?

The different elements for licensing animal research in the UK are laid out on the ASRU website.^11^ To get permission to do research on protected animals, which include “any living vertebrate other than man and any living cephalopod”,^12^ you need to be working at a licenced facility. The establishment needs to have named individuals in place responsible for veterinary care, animal welfare, information, compliance, and training and to comply with the ASRU (2014a) *Code of Practice* relevant for holding and using the animals located there. The institution will need to have a local Animal and Welfare Ethical Review Body (AWERB) whose responsibility is to ensure animal care, support staff, review procedures, and promote the 3Rs. You will need to apply for a personal licence, which in turn demands you have completed relevant ASRU Training Modules,^13^ themselves accredited by the Society of Biology. For procedures resulting in harm equivalent to or higher than that caused by expert insertion of a hypodermic needle you will also need a project licence. These cover programmes of research for up to 5 years, so can extend beyond 80 pages, explaining the purpose, place and plan of research, all experimental protocols, the numbers of animals used, the severity of procedures, any expected adverse effects, planned endpoints and methods of killing. You will need to propose a harm-benefit analysis of your work, which will be considered by the local AWERB, and evaluated by the ASRU Inspector, who will either grant permission to embark on this programme of work, or not.

In contrast, in the USA, only some animal research is covered by federal law and as indicated above animal research is regulated through a mix of legislated and non-legislated oversight. The amended 1966 Animal Welfare Act famously excludes legal protection for the most commonly used mammals in research: mice and rats. The initial assumption, under the 1966 Animal Welfare Act, was that all warm-blooded animals would be covered, unless specifically exempted – as in farm animals, understood only as food or fibre. The initial act covered “any live or dead cat, dog, hamster, rabbit, nonhuman primate, guinea pig, and any other warm-blooded animal determined by the Secretary of Agriculture for research, pet use or exhibition”.^14^ Yet, confirming the scope of animals included in the Act took over 30 years, with repeated attempts by US humane societies to push for the most inclusive definition. In the end, the 2002 amendment specifically excluded birds, rats of the genus *Rattus*, mice of the genus *Mus*, as well as all cold-blooded animals. The amendments were directed by budgetary constraints at the USDA, the agency charged with implementing the act, rather than by a specific ethical argument. There were insufficient resources made available to extend the record keeping and regulatory oversight that the act required for larger animals to the growing numbers of laboratory mice and rats in laboratory research, leaving many of these creatures in a legal limbo.

However, all US publicly-funded research does require adherence to *The Guide* introduced above, which operates through a “system of [both] self-regulation and regulatory oversight” (p.1). The US Animal welfare Act explains “Every institution that uses animals for federally funded laboratory research must have an Institutional Animal Care and Use Committee (IACUC). Each local IACUC reviews research protocols and conducts evaluations of the institution's animal care and use, which includes the results of inspections of facilities that are required by law”.^15^ The use of *The Guide* is mandated for federally funded research and advisory for other research. Learned societies, funders, and other organisations further extend responsibilities outside of these funding requirements and national contexts. Voluntary accreditation by AAALAC which is often valued by collaborators requires facilities to participate in their international animal care programme, based on ‘*The Guide’*, whilst also complying with local laws regulating animal research. There are fewer premises and projects excluded from law than first appear in the USA.

In addition, the technology of the licence, which is a central facet of the regulation of animal research in the UK, challenges some of the assumptions about the rigidity of centralised governance. As critical legal theorist Valverde (2003) suggests through reference to her work on cities, licences operate as complex and flexible forms of governing.^16^ In animal research, the licence externalises the state to manage the practices of laboratory animal research by delegating responsibility to different expert bodies, institutions and named individuals, supported by written guidance, published standards, external accreditation and inspection, and internal review. Licences, and related legal technologies like permits, are common techniques for balancing law and freedom within, and increasingly outside of, liberal societies. Here they seek to balance social recognition of animal sentience in law, which prevents cruelty, and utilitarian logics, which permit animal experimentation. The licences accrediting facilities, specifying standards, and authorizing individuals, both facilitate *and* regulate laboratory animal research. As Valverde suggests, the use of licences means actors are “not so much regulated as “responsibilized” to regulate themselves and the spaces they control” (2003, 49).

In many ways, this comparison demonstrates opposing positions in the location of institutional responsibility to permit animal research I introduced above. In the UK, this responsibility resides through law with central government; in the USA, much of the state's legal responsibility for animal research is discharged through local institutions. However, in practice both systems involve a mix of law and professional judgement, and central and local practices, in the discharge of responsibilities for permitting animal research. In the UK, licensing is a rather more flexible form of regulation than it first appears. Valverde provocatively suggests that licensing practices involve both “epistemological creativity” (2003, p26) and “a swamp of discretion” (Valverde & Weaver, 2015, p. 116) in relation to judgements of both competency and compliance. In the US, the oft-remarked legal absences for animal protections do exist, but these exclusions are rather more constrained than they first appear. Understanding the implications of this potential convergence requires further attention to the distribution of epistemic authority to shape animal research and care in practice.

### 3.2 Who sets standards for animal welfare?

A second contrast between US and European animal research is whether performance or engineering standards are seen as the best method for furthering laboratory animal welfare in practice. There has been relatively little attention given to the determination and use of standards in the social scientific literatures on laboratory animal research, though there is a related, but rarely linked, literature on the development of input versus output measures of animal welfare in farming (Velarde & Dalmau, 2012). In animal research, some countries favour engineering standards, with detailed guidelines for animal care methodologies and technologies, such as cage size; others prefer performance standards, where desired welfare outcomes are described, but methods flexible. The argument between standards can again be illustrated through a contrast between the UK and Europe – characterised as preferring engineering standards – and the USA – where performance standards are favoured. The adoption of these systems relates to the distribution of epistemic authority to speak for animals in practice. Performance standards require the considerable authority and discretion of veterinary professions in experimental settings, whilst engineering standards often represent the outcome of wider debates across different interests.

Engineering standards generally refer to those aspects of animal housing and care that can be specified numerically: including cage sizes, housing densities, temperature ranges, humidity levels and so forth. Engineering standards tend to be applied to facility design and animal accommodation but can also be found in the detailed parameters required of experimental protocols and reporting. The opening page of the Directive explains the role that minimum standards, common inspection and reporting requirements, and central repositories play in the governance of animal research in the EU. “It lays down minimum standards for housing and care, regulates the use of animals through a systematic project evaluation requiring *inter alia* assessment of pain, suffering distress and lasting harm caused to the animals. It requires regular risk-based inspections and improves transparency through measures such as publication of non-technical project summaries and retrospective assessment. The development, validation and implementation of alternative methods is promoted through measures such as establishment of a Union reference laboratory for the validation of alternative methods supported by laboratories within Member States.“^17^ Engineering standards are found widely throughout laboratory systems, for example, in ensuring biosecurity, health and safety or quality control through ‘good laboratory practice’ (World Health Organization, 2010). There are also many examples of engineering principles used within the laboratory animal sciences; for example, in tables on species and required cage sizes in the *UK Code of Practice* (2014b). The level at which standards should be set across Europe was a key point of discussion in the development of the EU Directive. Engineering standards are seen as playing a key role in assuring high standards of welfare across Europe. Countries that did not meet the new standards, for example increased cage sizes for mice, had a set time period to ensure they were compliant. However, member states were only allowed to adopt higher standards than new European ones if they were in their previous legislation. Engineering standards have become the preferred approach to many elements of European regulation, which has to manage a diversity of states. Engineering standards make compliance easier to evaluate, by setting baselines from which to judge contraventions and prevent countries erecting barriers to trade through setting higher standards.^18^

In contrast, performance standards refer to indicators of animal condition and behaviour thought to reflect their welfare, including growth, weight, aggression, reproductive performance, or the presence of disease. Performance standards are the preferred approach for evidencing and authorizing laboratory animal use and care in the USA. Performance standards were first described in the 7th edition of *The Guide* (National Research Council, 1996) and are defined in the American Association of Laboratory Sciences (AALAS) housing guide as follows:

“Performance criteria use the professional input and judgment of the laboratory animal veterinarians and the animal care staff – those individuals with the most intimate knowledge of the needs of the animals within their care. The performance approach defines an outcome in detail and provides the criteria for assessing that outcome. This approach does not limit the methods by which the outcome is achieved”^19^

The 7th edition of *The Guide* is critical of engineering standards, which it suggests “are sometimes useful to establish a baseline, but they do not specify the goal or outcome in terms of measurable criteria […] as do performance standards” (National Research Council, 1996, p.3). Their advocates argue performance standards are adaptable to circumstance and new scientific evidence and can be used by laboratory animal veterinarians to evaluate animal welfare through their professional experience and technical expertise. Engineering standards, they argue, are not always supported by scientific evidence and may be open to capture by vested interests. Conversely, critics of performance standards focus on the validity of assumptions made about how far professional veterinarians in different places are authorised to evaluate and uphold standards of animal welfare. They point to the lack of trust in local professional standards. They are also concerned that problems can only be identified after animal suffering is evidenced and point out that there are few funds to support the welfare science that would enable improvements to be evidenced in advance. They are also uneasy about what is already engineered into the system, for example, the fixed investments in animal housing, the standard operating protocols or notions of ‘historical control^’^, which limit local flexibility to achieve animal welfare outcomes.^20^

The preference for engineering or performance standards illustrates issues around the distribution of epistemic authority introduced earlier. This authority is more concentrated in the US system, in ways that both draw on and reinforce the professional standing of the role of the veterinarian. Epistemic authority is more distributed in the UK and Europe, where vets and animal care staff still play a key role, but where certain minimum protections are mandated through law and reinforced through inspection in ways that seek to smooth differences between member states. In practice, there is again some blurring between the two different approaches as European countries manage the tensions that come with harmonisation with some use of performance standards,^21^ and as *The Guide* is increasingly used to accredit overseas facilities through its minimum engineering standards.

These two factors can be used to see how the UK and USA constitute the regulation of animal research differently. They locate institutional responsibilities for permitting research across national and local bodies and authorise different professionals to identify and implement standards for animal welfare.^22^ In the next section, I explore how these patterns of research governance are embedded in the civic epistemologies in each nation, giving brief examples from earlier reviews of animal research, before charting how they are involved in efforts to extend international influence today.

## 4 Situating constitutions in animal research

### 4.1 Comparing civic epistemologies

Returning to Jasanoff (2005), we can suggest that different political systems, legal traditions, and administrative styles lead to different patterns of expert and public engagement in scientific decision-making. Britain has a common law, no written constitution, and an informal style of administrative decision-making, based on a flexible system of advisory committees and scientific inquiries. In the USA, there is a shared tradition of common law, but with a written constitution and a formal administrative style of decision-making built around a defined role for technical expertise. In Britain, decision-making proceeds on a case-by-case basis, usually overseen by a trusted individual asked to chair the process and author the subsequent report. Expert consultation is informal and public participation in decision-making is usually by invitation. In the USA, decision-making takes place within more formally organised institutional processes, based around the technical contributions of experts and the open participation of all interested groups. These processes are indicative of the more extensive ‘civic epistemology’ operating in the different countries – “contentious” in the USA and “communitarian” in the UK – which encapsulates the range of processes and traditions that shape scientific authority and public reasoning within these national political cultures (Jasanoff, 2005).

Table 2 can help understand the national civic epistemologies through which animal research is constituted within the USA and UK. In the US, styles of public knowledge-making are pluralistic, with competing interests arguing their case, as in the legal battles over the exclusions of the Animal Welfare Act. In the UK, knowledge-making is more centralised, within a service-based model, exemplified by the role of ASRU in licensing animal research under the ultimate authority of the Home Secretary. In the USA, courts are entrusted with ensuring public accountability, so arguments revolve around the legal standing of those attempting to get rodents included under the US Animal Welfare Act (Rollins, 2006). In the UK, accountability is relational, based around assumptions of delegation and trust, with Ministers held to account by the standards required by the office and the independent challenge of advisory bodies like the Animals in Science Committee. These broadly political and sociological differences help explain why institutional responsibilities for permitting research are located differently in the UK and USA.

**Table 2:** Comparative civic epistemologies.

	Britain “Communitarian”	USA “Contentious”
Styles of public knowledge-making	Embodied, service-based	Pluralist, interest based
Public accountability (basis for trust)	Assumptions of trust; relational	Assumptions of distrust; legal
Demonstration (practices)	Empirical science	Sociotechnical experiments
Objectivity (registers)	Consultative, negotiated	Formal, numerical, reasoned
Expertise (foundations)	Experience	Professional skills
Visibility of expert bodies	Variable	Transparent

Source: adapted from Jasanoff (2005, p.259).

**Table 3:** Situating variations in governance in national civic epistemologies.

		Institutional responsibility to permit research	
		centralised	distributed
Epistemic authority to shape research and care	concentrated		US research is authorised locally by the IACUC system, whilst epistemic authority is concentrated in professional veterinary and scientific roles, within a broadly contentious civic epistemology
	distributed	UK research is authorised centrally by the licensing system, whilst epistemic authority is distributed across the many actors who contribute advice to the definition of standards within a broadly communitarian civic epistemology	

However, the analysis by Jasanoff (2005) goes further, arguing there are different requirements for demonstrating scientific authority too, leading to epistemic differences in the use of science within the development of policy and the implementation of standards. In the USA, there is an emphasis on socio-technical experiments, whereas the UK has a more diffuse understanding of the role of empirical science in policy-making. This is the ‘cold American attitude’ in the opening quote. It can be seen in the prioritisation of different empirical practices in animal ethology, which have developed in the USA and Europe (Burkhardt, 2005; Haraway, 1989). Historically, in the USA, the discipline of ethology developed through controllable experimental practices on animals in captivity. In Europe and the UK, the discipline of ethology was more reliant on field-based observational studies. These disciplinary differences reflect divergent understandings of what is required for empirical verification in science, leading to different readings of the status of laboratory and field data (Kohler, 2002), which continue in different interpretations of the science underpinning behavioural studies and welfare research.

There are different registers of objectivity too. The USA demonstrates a preference for objective and numerical analytic tools, such as quantitative risk assessments, so will look for formal statistical reasoning in indicators of animal welfare before things like new enrichments or cage sizes are agreed. In this context, performance standards introduce an important role for professional judgements in policy contexts that would otherwise demand difficult to secure standards of scientific proof for action. Engineering standards may be too rigid and difficult to define within this epistemic framework; something which can be seen in the continued challenge to produce evidence for larger cages and enrichments for rodents (Balcombe, 2006). In the UK and Europe, these registers of objectivity are more open to different values, through negotiation, allowing for a wider range of positions from which to speak for animals. Notions such as animal integrity fail to fulfil US registers of objectivity and expertise, which seek formal reasoning and professional skills to close down scientific uncertainties. However, this is an acceptable term in European contexts, accorded status through experience as an object for further discussion (De Vries, 2006). There are also different definitions and assessments of animal pain between the USA and the EU, as well as divergence about the relevance of terms like normal animal behaviour, or the notion of a good life, to furthering debates about animal welfare (see for example Dawkins, 1980; Carbone, 2011). Although the EU policy environment is increasingly focused on building evidence, animal welfare is still recognised as a subjective judgement.

Finally, the contentious nature of American policy formation means the deliberations of expert bodies are made freely available. In the UK, despite a growing emphasis on openness around the uses and experience of animals in laboratory research (McLeod & Hobson-West, 2016), many aspects of policy deliberation, licensing practices and decision-making remain opaque to publics.^23^

In summary, the two variable dimensions to the regulation of animal research – the location of institutional responsibility and the distribution of epistemic authority – are configured in the UK and the USA in a way that reflects the civic epistemologies within these national contexts. Table 1 can be redrawn to include these additional contextual elements (see Table 3).

It is possible to illustrate this summary through returning to the recent reviews of US and UK animal research regulations. In the USA, this arena of science policy is characterised by an emphasis on the formal peer review of sound science, carried out by technically qualified experts, taking place in public, and reporting to advisory bodies that look for a balance between competing interests. The 2008 review of *The Guide* followed this model of scientific decision-making. This process depends and builds on shared norms of scientific evidence and review. However, it has limitations. It is susceptible to stasis in the face of divergent or unresolved evidence, as in struggles to build the evidence base for larger cage sizes, or when scientific and economic values seem incommensurable, as in debates over the inclusion of rodents in US law. In contrast, in the UK, definitions of objective science are based on the understanding of a more distributed empirical common knowledge, which is embodied by experienced individuals, who are often members of the Civil Service, who chair committees or inquiries, and whose job it is to speak for the public good. The House of Lords Select Committee on Animals in Scientific Procedures, which reported in 2002, and formed the basis of the UK's National Centre for the 3Rs, would be an example of this form of scientific advice operating in the UK (House of Lords, 2002). This process hinges on critical relational qualities of trust and delegation, consultation and negotiation. Yet, this reliance upon professional experience and assumptions of trust, rather than public transparency around scientific evidence and competing values, means it is susceptible to individual and institutional practices that may challenge wider social investments of trust (Lyons, 2011).

There are challenges to both systems, which are mitigated to some extent by their apparently opposite configurations of state power in research permissions and epistemic authority in research practice, and their location in specific civic epistemologies that shape political and public accountability. Jasanoff (2005) and Hilgartner et al., 2015 work on constitutional forms and civic epistemologies help to understand the origins and implications of these national contrasts. Yet, additional challenges emerge as they increasingly encounter each other, and the different local civic epistemologies, in geographical contexts outside of Europe and the USA.

### 4.2 Extending influence overseas

In this final section, I reflect on why these divergent frameworks have generated enough heat to be identified as a ‘cultural war’. Alongside attempts to categorise and harmonise regulations (Bayne & Turner, 2019; Demers et al., 2006), the meetings and journals of laboratory animal science have also seen a growing debate over competing global visions for laboratory animal research (Clark, 2007). As more countries introduce legislation that seeks both to protect animals and facilitate biomedical science, governments without long histories of animal research are searching for regulatory practices that fit local contexts and build international trust in their science and animal care. Countries like the UK and USA regularly send government delegations to inform these discussions and speak for their own regulatory preferences. Examples include the recent initiatives between ASRU and colleagues in the 10.13039/501100000617Foreign and Commonwealth Office (10.13039/501100000617FCO) with the aim of “supporting our partners in embedding the 3Rs in their science, thus supporting collaborations between UK and Chinese scientists” (ASRU, 2014b, p. 6) and the ongoing role of AAALAC in accrediting overseas institutional facilities.

These initiatives are motivated by a desire to extend animal protection and concerns about losing competitive advantage in international research. The general argument goes that promotion of regulation for animal research in emerging knowledge economies helps support animal welfare and ensure the quality of science. The specific forms that regulation takes then shapes the flow of research, resources, equipment, consultancy, and collaborations. It has the effect of connecting, or disconnecting, new research centres from colleagues in the UK or USA. These two points were made in conversation with a regulator in 2010.^24^ First, they make the argument linking animal welfare to good science and seeking to avoid research with lower scientific, and welfare standards. Second, they show how standards are part of the struggle for international influence between established centres of science.

*I think some of the concerns are to do with the emerging countries that are really sort of going to siphon off research. So I think people are concerned […] to try and make sure that international standards are in place so that there is a kind of level that has to be met, so that you are assured that the data that's being generated is actually valid and can actually be used. So, I think there's that kind of concern. But then I think there's also key people that are involved, that it’s almost like a bit of a power struggle that's going on, that we will kind of stamp our mark on these guidelines and standards and we will make sure that it’s done in a UK like way, or we'll make sure it's done in a US kind of way.*

The differences between UK and US styles of regulation are part of the pursuit of influence and spatial connection in the emerging geographies of science. State strategies for research governance within new knowledge economies have been outward-looking, multi-layered, and focused on biotechnological innovation (Salter, 2009; Ong & Chen, 2010). Where regulations for international biomedical research have been developed, there has often not been the anticipated regulatory rush to the bottom, providing places for unethical scientific experimentation that critics assumed.^25^ Rather, countries like Singapore have pragmatically sought the most efficient regulatory mechanisms to connect emerging research to global standards, create an attractive environment for overseas researchers, build trust through familiar laboratory protocols, and enable licensing of therapeutic products in the valuable US market. The adoption of regulatory practices from the UK, Europe or USA does influence subsequent patterns of research collaboration, scientific migration, and intellectual investment. This is not because one system is seen by internationally mobile scientists as necessarily better than any other, but because they are familiar, so are more trusted by those collaborating internationally and more comfortable for those relocating overseas.

However, the cultural embeddedness of regulation outlined above does raise concerns that practices which are constituted in one national context may have different outcomes in another. This can be illustrated through a brief example of how UK and US regulations have been used in Singapore. Here responsibility to authorise animal research is centralised and follows many elements of the UK system, in part reflecting forms of civil administration introduced when Singapore was a UK Crown Colony in the 1940s and 50s. However, the day-to-day practice of laboratory animal care follows the USA in adopting performance standards for animal welfare, and many veterinarians working in Singapore come from the USA. In theory, the epistemic authority for the operation of animal welfare is then concentrated in these individuals. However, the absence of an established tradition of veterinary care and animal research in the city state, and the lack of an active public sphere mobilised around animal protection, creates a civic epistemology that means this authority is more limited in practice.

The two key dimensions of regulation can be traced again, however, in Singapore the authority to permit research and shape research are both centralised. The concentration of both the responsibility to permit research and the epistemic authority for the operation of research and care brings risks. It means the regulation of animal research is more likely to be closely allied to changing strategies for state innovation in biotechnology, rather than by animal welfare. One vet, previously employed in the USA and working in Singapore at the time of our conversation, suggested their ability to exercise discretion differed in their work in Singapore: “the format of it is a little bit different. I notice things here tend to follow more like bulleted items. And there tends to be stuff that gets dropped out or things maybe don't get enforced as much”. The outcomes of regulatory practice depend on their local recontextualisation.

The geographical extension of regulations for animal research is taking place in contexts in which styles of public knowledge-making are different from those in either Western Europe or the United States. Standards, guidelines, and other licensing practices may not be understood or applied in the same way outside of the civic epistemologies in which they were forged. The application of discretion in either permitting research or setting standards can be uncertain when new knowledge economies lack historic experience in the veterinary sciences, regulation is prompted as much by practical as ethical concerns, and local civic epistemologies are opaque to those unfamiliar with them. The use of regulatory techniques within contexts in which civic epistemologies are different means they have different outcomes. The challenges that emerge can be summarised in a final table (see Table 4).

**Table 4:** Comparing key variables in the regulation of animal research in context.

		Institutional responsibility to permit research	
		centralised	distributed
Epistemic authority to shape research and care	Concentrated	Risks of rigidity, inadequate challenge, or state control	Compromise between institutional flexibility and professional control supported by performance standards
	distributed	Compromise between institutional control and distributed expertise facilitated by use of engineering standards	Risk of ambiguity, lack of transparency, or regulatory capture

These local civic epistemologies are dynamic, complex, and changing rapidly as those individuals, institutions and ideas associated with the development of laboratory animal research travel. However, arguments continue over how to improve animal science and welfare (Bayne & Turner, 2019) and as the UK faces renewed challenges in reconstituting its national regulations and international connections, the potential for a resurgent culture war remains.

## 5 Conclusions

In this paper, I have suggested international differences in the constitution of animal research can be understood through the interplay of two key variables: i) the *location of institutional responsibility to permit research* and ii) *the distribution of epistemic authority to shape research and care*. I have illustrated this through exploring the legal frameworks for ethical review in the UK and USA and the differences between performance and engineering standards for animal welfare. I have also argued this framework matters for two reasons. First, it is useful retrospectively for identifying different constitutional forms around animal research, locating these within histories and geographies of public knowledge-making, understanding their potential vulnerabilities, and examining the relationships between them. Even with increased mobility in science, research carried out by Friese et al. (2019) indicates that national identity and civic epistemology are key factors in explaining international differences in laboratory animal science and care. Secondly, it can be used prospectively. As choices open up around how to regulate animal research globally, this analysis shows how different forms of regulation already reflect cultural assumptions about political and epistemic authority, so helping identify effective routes for improving science, ethics, and care in different contexts. Locating the wider political, social, and epistemic commitments involved in the constitution of animal research can both indicate the choices available to the UK in reconstituting animal research following Brexit and the challenges in changing forms of regulation that are so deeply cultural embedded.

This account also connects to the academic literatures increasingly engaging with the scientific, policy, and spatial practices of laboratory animal research. Scholars in the humanities and social sciences are increasingly trying to scale up accounts of how responsibility and care operate in laboratory animal research from discussions of corporeal encounters based on co-presence (Greenhough & Roe, 2011), to the development and maintenance of institutional cultures of care (Gorman & Davies, 2020), through to national frameworks (Friese et al., 2019) and international exchange (Druglitrø & Kirk, 2014). Drawing things together at international scales is complex but important. It helps to show that even when languages converge, practices may not. For example, institutional strategies for a ‘culture of care’ are a regulatory requirement in Europe and the UK but are viewed as a way of preventing burdensome central regulation in the USA (Davies et al., 2018). Conversely as indicated above, there are times when policies diverge, but are closer in practice than might be expected. Attempts to build social and ethical theory on the basis of ethnographic accounts of laboratory animal science and care also have to grapple with these differences. Increasing spatial connectivity is not the same as creating and implementing a global vision for laboratory animal science or care. Scale is not constituted through a single scientific outlook, but rather through local flexibilities with the potential to make data meaningful (Leonelli, 2013) and care effective in experimental situations. Furthering laboratory animal care may be sought as much through understanding local civic epistemologies, identifying the operation of administrative knowledges, and deepening professional capacities in emerging bioeconomies, as by globalising standards (Niemi & Davies, 2016).

However, it is important to acknowledge the limitations of this paper's focus on the constitutional mechanisms for regulating animal research. The overall aims of regulation do still vary from place to place, with significant consequences for animal use and care. At the end of this long analysis of regulatory responsibilities it is still not possible to answer the question implied in the opening quote from ten years ago clearly: is it really the case that in all three situations in Canada, the USA, and Europe the animals appear to be doing well enough? The increasingly standardised cage structures that shape animal care and the demands of international collaboration that require ethical review do likely mean that the mice have a similar experience. Given enrichment and the opportunity to thermoregulate in their housing perhaps the mice are doing well enough, though the conversation about links between welfare and data continue (Garner et al., 2017). However, for other species like primates, international differences between conventional housing are still striking. Informal conversations reveal anecdotal evidence of individual researchers both moving to places where their work is facilitated and then moving back following experience of lower animal welfare standards. To answer that question fully, the analysis in this paper would need to be supplemented by comparative animal welfare science on the experience of animals and cross-cultural social science that included discussion of different species. I would warmly welcome this further work. However that research would still need to be embedded in a framework for understanding the differences, convergences, and competition between changing constitutions of animal research if its outcomes sought to inform animal welfare, rather than add fuel to an ongoing cultural war.

## Funding

This research was funded in whole, or in part, by the 10.13039/100010269Wellcome Trust [205393/Z/16/Z, 2017–2023]. For the purpose of open access, the author has applied a CC BY public copyright licence to any Author Accepted Manuscript version arising from this submission. The work underpinning this also supported by an 10.13039/501100000269ESRC Impact Accelerator Award [2015–6], a further Wellcome Trust small grant [104339/Z/14/Z, 2014–2015], and an ESRC Research Fellowship [RES-063-27-0093, 2007–2010].

## Data availability

Due to the sensitive nature of the research area, and in accordance with conventions and offers of confidentiality made at the time data was collected, I do not have permission to make the interview transcripts and other observational data from this research publicly available.

## Footnotes

1 The primary focus of this paper is on UK legislation and the 1986 UK Animals (in Scientific Procedures) Act (also known as A(SP)A), but the recent history of these regulations cannot be understood outside of EU law. A(SP)A was a key influence on the drafting of the EU Directive from 2008 onwards, the EU Directive was transposed into UK legislation in 2013, and following Brexit in 2020 all of appendices referring to the EU Directive were fully incorporated into A(SP)A through secondary legislation.

2 International Council for Laboratory Animal Science.

3 International Council for Harmonisation of Technical Requirements for Pharmaceuticals for Human Use.

4 This account has been developed through three bodies of empirical work engaging with key events and actors in the national and international regulation of animal research from 2007 to the present. First is work completed 2007–2011 tracing collaboration in international projects phenotyping mice (Davies, 2013). This involved interviews with researchers in the UK and USA and included visits to some of these review meetings as well as other countries in Europe, North America, and South-East Asia. All interviews were carried out with the written consent of participants, who were offered confidentiality, so all names and personal details have been removed. Second is my role as a lay member of the UK Government's Animals in Science Committee (2013–2019), where I chaired the subgroup reviewing UK Harm-Benefit Analysis (Davies, 2018). My reflections here are based on personal readings of the final versions of published policy documents and do not reflect the work or opinions of the committee. Thirdly, collaborative work within the Animal Research Nexus programme (2017–2022) has sought to build connections between academic research and policy on the social aspects of animal research (Davies et al., 2016, 2020) and locate animal research within the historical trajectory – or nexus – of national cultures and constitutional forms (Kirk, 2017).

5 These are commonly referred to as institutional animal care and use committees (IACUCs) in the USA and animal welfare and ethical review boards (AWERBs) in the UK.

6 The minutes of the independent UK Animals in Science Committee indicate that it started an exercise to review international policies for regulating animal research, but this was not completed, see https://www.gov.uk/government/organisations/animals-in-science-committee/about/membership#minutes, last accessed 02/08/2021.

7 The most recent commissioning letter for work by the UK Animals in Science Committee talks about collaboration to develop a “suitable metric for the delivery of protecting animals in science” https://www.gov.uk/government/publications/ministerial-commission-of-work-for-the-animals-in-science-committee-2020-to-2021, last accessed 02/08/2021.

8 I have written about this in relation to the different understandings of a ‘culture of care’ in an earlier article. In the UK context, the definition of a culture of care emphasizes the character of local establishments within a framework of national governance: “A good culture of care is an environment which is informed by societal expectations of respectful and humane attitudes towards animals used in research. Each establishment will have its own way of conveying its culture of care. However, all establishments are subject to similar governance and legal responsibilities under ASPA to deliver humane care” (ASRU, 2015). However, in the USA, a culture of care is counterpoised to a culture of control. Klein and Bayne (2007) discuss their aspiration for “establishing a culture of care, conscience, and responsibility” through a strategy “to prevent burdensome regulations”.

9 Estonia, Germany, Portugal, Romania, Slovakia, and Spain.

10 http://europa.eu/rapid/press-release_MEMO-18-4486_en.htm, last accessed 02/08/2021.

11 https://www.gov.uk/guidance/research-and-testing-using-animals#applying-for-licences, last accessed 02/08/2021.

12 In the UK, protected animals under A(SP)A are “any living vertebrate other than man and any living cephalopod” https://www.legislation.gov.uk/ukpga/1986/14/section/1, last accessed 02/08/2021.

13 http://www.learningcurvedevelopment.co.uk/courses/home-office-modules-1-4/, last accessed 02/08/2021.

14 https://web.archive.org/web/20130327214345/http://www.nal.usda.gov/awic/pubs/AWA2007/intro.shtml, last accessed 02/08/2021.

15 https://web.archive.org/web/20090416104435/http://www.nal.usda.gov/awic/legislat/usdaleg1.htm last accessed 02/08/2021.

16 The work of Valverde explores how the licensing of drinking, food, zoning and planning shapes urban form and practice. She explores how licences and permits operate in cities as flexible forms of governing. They can control access to something (e.g. alcohol) or enable something to happen (e.g. building); they can be technologies of security and control (e.g. clubs or guns licences); they can certify skills to carry out activities (e.g. driving licences); they can also be used to ration access to scarce resources or to raise funds for services (e.g. parking permits). Her work emphasizes the discretionary nature of licensing, its relation to different forms of knowledges, and the interplay between law, science and the open-ended or “unfinished” forms of administrative knowledge and bureaucratic expertise through which licencing operates (Valverde (2003), 22). These insights can be extended to laboratory animal research, where licensing is permissive, changing over time, varying over space, inherently heterogeneous and so also involves considerable discretion in application. Her work can also be used to explore how licences shape our relationships with animals in other domains, such as through the licences required to run zoos, breed or board cat and dogs, keep wild animals, which give shape to the boundaries between private activities and public sensibilities, and are administered by local authorities in the UK. Licences are not required for many activities using animals, including farming, but licences are required for transport and animal shows, where the emphasis is on controlling disease and protecting public health.

17 See https://ec.europa.eu/environment/chemicals/lab_animals/legislation_en.htm, last accessed 02/08/2021.

18 For the full set of Directives, including information on procedures, reporting, and housing (including cage sizes), see https://eur-lex.europa.eu/LexUriServ/LexUriServ.do?uri=OJ:L:2010:276:0033:0079:EN:PDF, last accessed 02/08/2021.

19 See https://www.aalas.org/about-aalas/position-papers/determining-laboratory-animal-housing-standards#.VkmzFL_YQlM, last accessed 02/08/2021.

20 These observations are taken from closed policy debates and open discussion at the National Academy of Science in Washington for the review for the 8th edition of *The Guide* (see National Research Council, 2011a).

21 The ASRU (2014a)*Code of Practice for the Housing and Care of Animals Bred, Supplied or Used for Scientific Purposes* does include specific reference to performance standards for those aspects of UK housing and care that exceed European requirements.

22 A full account of the differences between UK and US animal research would additionally require consideration of cultural attitudes to animals as well. The aim of this paper is to offer a framework for understanding relations between science, law, and governance. As I indicate in conclusions, I hope this might be elaborated with further research on the position of animals within different contexts in the future, but this is beyond the scope of this current paper.

23 Due to the centralised records of potentially sensitive commercial information and the security environment when it was drafted, the UK Animals (Scientific Procedures) Act 1986 (ASPA) contains a so-called ‘secrecy clause’. Section 24 is a statutory prohibition on disclosure, protecting information received in confidence in connection with the regulatory activities of ASPA at the Home Office. There are long term plans to review Section 24 of ASPA.

24 Given the small number of regulators in the country in which this interviewee is based, their location has to remain unnamed to preserve anonymity.

25 Though there are still contentious practices, such as the genetic modification of primates and animal experiments on whole body transplants, that are carried out in some places, including China, that would not be authorised in other places, like the UK or Europe.
